# An Intelligent Condition-Monitoring Framework for Alkaline Water Electrolyzers Based on Hybrid Physics-Informed Health Indicators

**DOI:** 10.3390/s26041090

**Published:** 2026-02-07

**Authors:** Jie Liu, Zhiying Wang, Tingting Ma, Xinyue Chen, Zihao Wang, Chao Huang, Yiyang Dai

**Affiliations:** 1 Xinjiang Chemical Engineering Design & Research Institute Co., Ltd., Urumqi 830010, China; 15739530259@163.com (J.L.); wangzhy1215@163.com (Z.W.); m15899106271@163.com (T.M.); 18690969510@163.com (X.C.); 18129345757@163.com (Z.W.); 2 School of Chemical Engineering, Sichuan University, Chengdu 610065, China; 2020223070046@alu.scu.edu.cn

**Keywords:** alkaline water electrolyzer, health indicators, condition monitoring, computational fluid dynamics, machine learning, predictive maintenance, intelligent sensing

## Abstract

**Highlights:**

**What are the main findings?**

- A hybrid physics-informed machine learning (ML) framework is proposed for constructing Health Indicators (HIs) and enabling intelligent condition monitoring of Alkaline Water Electrolyzers (AWEs).
- Trained on a CFD-generated dataset, a Multilayer Perceptron (MLP) model achieves 90.43% accuracy in real-time health state classification, serving as an effective intelligent monitoring agent.

**What are the implications of the main findings?**

- The proposed methodology provides a practical solution for predictive maintenance of AWEs operating under volatile renewable energy, enhancing system safety and reliability.
- It demonstrates the significant potential of combining mechanistic models with machine learning for intelligent monitoring in complex industrial systems where sensor data is limited.

**Abstract:**

Alkaline Water Electrolyzers (AWEs) are critical for green hydrogen production but face operational risks due to volatile renewable energy inputs. This study proposes an intelligent condition-monitoring framework that leverages a hybrid physics-informed machine learning (ML) methodology to construct Health Indicators (HIs). The core innovation lies in addressing the challenge of inaccessible internal states. First, a high-fidelity Computational Fluid Dynamics (CFD) model is developed and experimentally validated, serving as a physics-informed data generator to simulate multiphysics behavior under various operating and fault conditions. From this reliable simulation basis, a comprehensive dataset is produced, and eight key operational parameters are derived as HIs. This dataset is then used to train and benchmark three ML models for rapid health state classification. The Multilayer Perceptron (MLP) model achieves superior performance with 90.43% accuracy, effectively translating the validated physical understanding into a fast, deployable intelligent monitoring agent. This work presents a viable pathway for constructing reliable HIs and implementing AI-enhanced condition monitoring for AWEs, contributing to safer and more efficient green hydrogen production.

## 1. Introduction

The global imperative to decarbonize energy systems has driven growing interest in green hydrogen as a sustainable energy carrier. Produced via water electrolysis powered by renewable energy sources (RES), green hydrogen supports deep emission reductions across transportation, industry, and power generation sectors [1,2,3]. Among electrolysis technologies, Alkaline Water Electrolyzers (AWEs) are the most mature and commercially deployed option for large-scale production due to their low cost, durability, and reliance on non-precious metal catalysts [4,5,6]. Consequently, AWEs are expected to form the backbone of the emerging green hydrogen economy.

However, coupling AWEs with intermittent RES introduces operational challenges. Fluctuating power input forces AWEs to operate under dynamic and off-design conditions, accelerating degradation and potentially triggering gas crossover, electrode and separator deterioration, thermal instability, and pressure excursions, which compromise efficiency, safety, and asset integrity [7,8,9,10]. These risks highlight the urgent need for intelligent, real-time health monitoring and predictive maintenance, aligned with the industrial shift toward Prognostics and Health Management (PHM) and condition-based maintenance (CBM) [11,12,13]. In this context, Health Indicators (HIs) provide compact representations of system condition, while Digital Twins (DTs) integrate physics-based models and data analytics to enable diagnosis and prediction [14]. For AWEs, effective HIs should combine measurable external variables with internal state information linked to degradation physics, but many internal states—such as local electrolyte concentration, gas holdup, or membrane stress—are inaccessible to conventional sensors.

Two complementary paradigms address this challenge: physics-based modeling and data-driven machine learning (ML) [15,16,17]. High-fidelity Computational Fluid Dynamics (CFD) enables detailed analysis of multiphysics phenomena in AWEs, including two-phase flow, current density distribution, temperature gradients, and mass transport [18,19]. When experimentally validated, CFD can generate synthetic datasets covering nominal and fault-induced conditions difficult to reproduce safely in experiments [20,21] supporting studies of flow patterns, bubble dynamics, and performance sensitivity [22,23]. However, CFD alone is computationally intensive and unsuitable for real-time monitoring. ML methods, in contrast, efficiently learn nonlinear relationships among operational variables, enabling fast online inference [24,25,26,27]. Integrated CFD–ML approaches have been explored to build surrogate models for performance prediction and optimization [28], but existing works primarily target a limited set of outputs or design efficiency, often lacking interpretability and applicability for PHM.

While recent studies have integrated computational fluid dynamics (CFD) and machine learning (ML) for AWE analysis—such as Sirat et al. [28] for performance enhancement and Bai et al. [20] for simulating flow and electric fields—they primarily aim at predicting or optimizing specific physical parameters. In contrast, this study proposes a novel physics-informed health indicator (HI) construction framework for system-level condition monitoring and health state classification. The core methodological innovation addresses the challenge of internal state inaccessibility by translating measurable operational data into actionable health insights for predictive maintenance. To this end, a novel framework founded on the integration of mechanistic CFD modeling and data-driven ML is developed. The foundation of this framework is an experimentally validated CFD model, which ensures the physical fidelity of the generated training data. The core contributions are threefold:

1. Development of a Systematic HIs System for AWE: A semi-quantitative health evaluation framework is established based on common AWE failure modes, defining health classes (from excellent to poor) based on thresholds for eight key operational parameters encompassing efficiency, safety, and stability.
2. Generation of a Physics-Informed and Experimentally Validated Dataset: A 2D multiphysics CFD model is developed and experimentally validated. This validated model is then employed to conduct parametric sweeps, creating a comprehensive and physically reliable dataset for ML training.
3. Benchmarking of ML Algorithms for Intelligent Condition Monitoring: Three distinct ML approaches—Polynomial Regression, SVM, and MLP—are implemented and rigorously compared for the task of classifying the health state of the AWE into the predefined categories. The optimal model is identified based on accuracy, robustness, and computational efficiency.

By leveraging the complementary strengths of physics-based simulation and statistical learning, this hybrid methodology delivers a semi-quantitative health evaluation tool effective for condition monitoring under steady-state or slowly varying operating conditions. It represents a step towards intelligent PHM for electrolysis systems, ultimately contributing to safer, more efficient, and more economical green hydrogen production.

The remainder of this paper is structured as follows: Section 2 details the comprehensive methodology, including the overall framework, the development of the multiphysics CFD model for HI data generation, and the configuration of the ML algorithms for intelligent monitoring. Section 3 presents the case study, describing the specific geometric and operational parameters of the AWE system under investigation. Section 4 presents the results and discussion, first validating the reliability of the CFD-generated dataset and then analyzing and comparing the performance of the different ML models. Finally, Section 5 concludes the paper by summarizing the principal findings, discussing the implications for industrial application, and outlining promising directions for future research.

## 2. Methodology for the Intelligent Condition-Monitoring Framework

**Figure 1:**
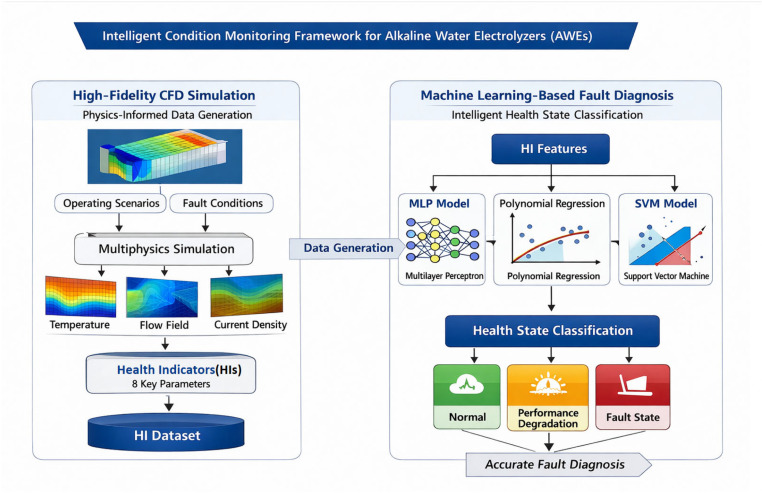
Overall framework of the hybrid physics-informed ML approach for intelligent AWE condition monitoring.

The proposed methodology for developing an intelligent condition-monitoring system for AWEs follows a structured two-stage hybrid framework, as illustrated in Figure 1. It is designed to systematically define measurable HIs, generate labeled data reflecting the underlying physics, and develop a fast inference model for online deployment.

### 2.1. Definition of the AWE HIs System

The cornerstone of an effective condition-monitoring system is a suite of quantifiable HIs that exhibit high sensitivity to incipient system degradation and failure modes [29]. The development of the HIs system began with a thorough analysis of common faults and failure mechanisms in AWEs [30,31,32], as summarized in Table 1. These faults, such as gas crossover, electrode corrosion, and overheating, manifest as observable deviations in key process parameters.

The selection of Health Indicators (HIs) in this study follows three guiding principles:

1. Physical relevance to dominant AWE degradation and failure mechanisms.
2. Measurability or inferability under industrial operating conditions.
3. Sensitivity to off-design and fault-related operating regimes.

Based on these criteria, eight key operational variables were identified as HI candidates, including Cell Voltage (V), Current Density (A/m^2^), Operating Temperature (°C), System Pressure (atm), Electrolyte pH/KOH Concentration (M), Bubble Velocity (m/s), Coulombic Efficiency (%), and Power Load (W).

These variables provide complementary information on electrochemical performance, thermal behavior, mass transport characteristics, chemical stability, and operational safety. To facilitate a structured health assessment, the selected HI candidates are further organized into three health-related dimensions—efficiency and consistency, power regulation flexibility, and gas purity—which together constitute an integrated AWE health indicator framework.

**Figure 2:**
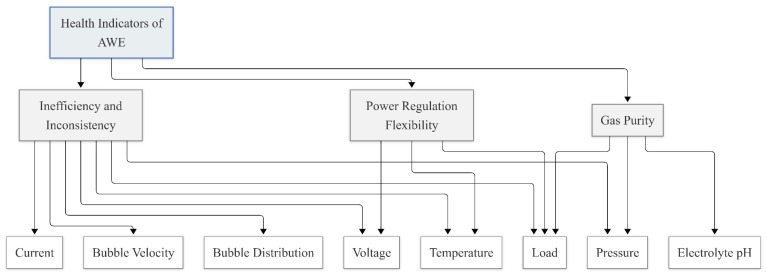
HIs for AWE condition monitoring (note that Coulombic efficiency is a derived indicator inferred from current and gas evolution behavior).

The conceptual mapping between operational variables, health dimensions, and the overall health state evaluation is illustrated in Figure 2.

To translate these continuous parameters into actionable diagnostic information, a semi-quantitative evaluation framework was established. For a given operating condition, each of the key parameters is evaluated against the predefined threshold ranges in Table 2 to determine its individual health sub-state. The overall health state of the AWE is then assigned as the most severe sub-state among all parameters, following a conservative logic similar to multi-dimensional risk assessment. The threshold values are not arbitrarily assigned; they are determined through a combination of theoretical electrochemical constraints, validated CFD-based simulation results under both nominal and fault-induced conditions, and operational expertise, enabling systematic differentiation between normal operation, progressive performance degradation, and high-risk fault regimes.

Although eight HIs were initially examined, only six representative indicators are retained for threshold-based classification in Table 2. This reduction is motivated by the intrinsic coupling among cell voltage, current density, power load, and efficiency, particularly under variable-power and load-following operating conditions typical of renewable-driven AWEs. Under such conditions, fixed thresholding of voltage or current alone cannot reliably distinguish between normal operational variability and genuine degradation or fault behavior. Including all coupled variables would therefore introduce redundancy without improving diagnostic discriminative capability. Consequently, parameters exhibiting relatively independent physical significance and higher sensitivity to fault-related deviations were prioritized to enhance the robustness and interpretability of the semi-quantitative health evaluation.

This HIs system provides the essential “vocabulary” for health assessment. The subsequent steps aim to build a model that can automatically assign these health-state labels (A–D) based on a subset of easily measurable inputs.

**Figure 3:**
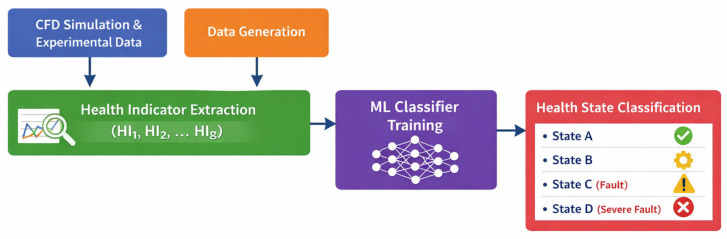
Conceptual workflow of the HI-based monitoring system for AWEs, showing CFD/experimental data, HI extraction, MLP classifier, and health state classification.

To further clarify the relationship between the defined health indicators and the machine learning classifier, a conceptual workflow is illustrated in Figure 3. This diagram depicts the process from CFD-based data generation, HI extraction, to the MLP classifier for health state prediction. In addition, feature importance analysis using SHAP values is integrated, highlighting the contribution of each HI to the classification of all health states. This enhancement provides both a clear visual mapping of the methodology and interpretable insights into model decision-making.

### 2.2. Physics-Informed Data Generation via CFD Modeling

To generate the labeled dataset required to train the monitoring model, a high-fidelity, physics-based CFD model of a generic AWE unit cell is developed. This model serves as a physics-informed data generator, simulating the complex, coupled electrochemical, thermal, and fluid dynamic processes to compute all eight HIs under a wide range of prescribed operating conditions.

It should be noted that the present CFD model adopts a two-dimensional representation and a simplified bubble size assumption. These choices are made to balance physical fidelity and computational efficiency, enabling large-scale parametric data generation for health indicator development. While three-dimensional bubble interactions and bubble size distributions may influence local flow structures, the adopted model is sufficient to capture the dominant coupled electrochemical–transport trends relevant to relative health state discrimination. Future work will extend the framework to incorporate 3D effects and population balance models as higher-fidelity experimental data become available. All simulations in this study were conducted using the commercial finite element software COMSOL Multiphysics (Version 6.1).

#### 2.2.1. Electrochemical Reaction Fundamentals

The electrolysis reaction in an AWE cell is governed by the decomposition of water into hydrogen and oxygen under an applied direct current, as expressed by the overall reaction:

H_2_O → H_2_ + O_2_ (1)

This occurs via two half-reactions at the electrodes immersed in an alkaline electrolyte (typically KOH solution). At the cathode, the Hydrogen Evolution Reaction (HER) takes place:

2H_2_O + 2e^−^ → H_2_ + 2OH^−^ (2)

At the anode, the Oxygen Evolution Reaction (OER) occurs:

2OH^−^ → O_2_ + H_2_O + 4e^−^ (3)

#### 2.2.2. Model Geometry and Mesh Strategy

A common approach involves constructing a simplified 2D geometry representing a symmetric cross-section of a unit cell, comprising the electrode compartments and a separating diaphragm. The geometry is discretized using a structured or mapped mesh to balance accuracy and computational cost. Critical regions, especially the electrode surfaces where bubbles nucleate and reactions occur, require mesh refinement (e.g., boundary layers) to accurately capture steep gradients in species concentration, current density, and gas volume fraction.

#### 2.2.3. Mathematical Formulation and Governing Equations

The CFD model was implemented using the “Water Electrolyzer” interface in COMSOL, which couples multiple physics interfaces. The core mathematical descriptions include the solution of the governing equations for conservation of mass, momentum, species, and charge, along with the Butler-Volmer electrochemical kinetics. Key constitutive relations and corrections are as follows:

1. Electrochemistry and Charge Transport

The local current density distribution is governed by Butler-Volmer kinetics [33]. The presence of generated gas bubbles significantly affects the system by reducing the effective area for reaction and charge transport. To account for this, the effective electrolyte conductivity *σ*_*l*,*eff*_ and the effective exchange current density *i*_0,*eff*_ are corrected using the Bruggeman correlation, which is standard for porous media and two-phase flows in electrochemical cells [34,35]:

(4) $$\sigma_{l,eff}\;=\;{\left(1-\phi_{d}\right)}^{1.5}\sigma_{l}$$

(5) $$i_{0,eff}=\;(1-\phi_{d})i_{0}$$

where *ϕ_d_* is the local gas volume fraction, and *σ_l_* and *i*_0_ are the conductivity and exchange current density of the bubble-free electrolyte, respectively.

2. Multiphase Flow Dynamics

The bubbly flow of H_2_ and O_2_ was simulated. within the electrolyte channels is simulated using the Euler-Euler module [36], which is well suited for modeling dispersed gas phases in liquid electrolytes. In the present framework, gas bubbles are represented using a constant, representative diameter, serving as a closure assumption to enable efficient simulation of gas–liquid momentum exchange under varying operating conditions. This treatment focuses on capturing physically consistent trends in two-phase transport behavior rather than resolving detailed bubble population dynamics.

The momentum exchange between the gas bubbles and the liquid electrolyte includes a bubble dispersion force *F_BD_* to model the turbulent dispersion of bubbles, a critical phenomenon in electrolyzer flows [37]:

(6) $$F_{BD}\;=\;-\phi_{d}\rho_{l}\frac{K_{g}}{d_{b}}\left|u_{slip}\right|{\nabla \phi }_{d}$$

where *ρ_l_* is the liquid density, *K_g_* is the dispersion coefficient, *d_b_* is the bubble diameter, and *u_slip_* is the slip velocity between phases.

3. Mass, Species, and Energy Transport

Coupled equations for continuity, species conservation (K^+^, OH^−^), and energy balance are solved to obtain spatial distributions of electrolyte concentration and temperature, which in turn influence reaction kinetics and material properties.

4. Numerical Methods and Model Purpose

The equations are solved using a segregated steady-state solver on a structured/polyhedral mesh. Convergence is monitored via residuals of all governing equations (<10^−6^). The model is designed primarily as a physics-informed data generator for constructing health indicators and training ML classifiers, providing accurate global trends while enabling efficient exploration of multiple operating and fault scenarios.

#### 2.2.4. Parametric Sweep for Dataset Generation

To create a comprehensive dataset that populates the health state space defined by the HIs system, a systematic parametric sweep is performed. Key operational input variables-such as cell voltage, operating temperature, system pressure, and electrolyte concentration-are varied across predefined ranges that encompass both normal and fault-inducing conditions. For each simulated steady-state operating point, the values of the eight target HIs are extracted. Each data sample is then automatically labeled with a health state (A–D) by applying the threshold rules from the HIs system (Table 2), resulting in a physics-consistent, labeled dataset for ML

This process yields a physics-consistent, labeled dataset (*U*, *T*, *p*, *c_KOH_*, …) → (*HI*_1_, *HI*_2_, …, *HI*_6_, *HealthState*) that forms the basis for training the data-driven monitoring model.

### 2.3. ML Model Development for Intelligent Inference

The computationally intensive CFD model is unsuitable for real-time deployment. This phase focuses on distilling the physical knowledge encoded in the CFD-generated dataset into a fast and accurate data-driven surrogate model for online intelligent monitoring.

#### 2.3.1. Data Preprocessing and Feature Engineering

The raw dataset $D={\left\{x_{i},y_{i}\right\}}_{i=1}^{N}$ from CFD simulations is preprocessed. Here, **x***_i_* is a vector containing the operational input parameters and potentially derived features, and *y_i_*∈{*A*,*B*,*C*,*D*} is the health state label. Common preprocessing steps include:

1. Handling Missing Values: Removing or imputing samples where CFD simulations failed to converge.
2. Normalization/Standardization: Features often have different scales (e.g., volts, °C, atm). Min-max normalization or z-score standardization is applied to improve model convergence and performance. Min-max normalization scales a feature x*x* to the range [0, 1]:
  (7) $$x_{norm}=\frac{\left(x-min\left(x\right)\right)}{\left(max\left(x\right)-min\left(x\right)\right)}$$
3. Feature Selection/Construction: The input feature vector *x* is constructed from the most informative and easily measurable parameters. This typically includes the swept input variables (e.g., voltage, temperature) and key calculated outputs from the model that have strong correlations with the health state.

#### 2.3.2. Model Training and Benchmarking Strategy

The preprocessed dataset is split into training, validation, and test sets. Multiple ML algorithms are benchmarked for the multi-class classification task (predicting health state A–D). Common choices include:

- Polynomial Regression: Serves as an interpretable baseline. The model’s capacity is controlled by the polynomial degree.
- SVM: A robust classifier effective in high-dimensional spaces. Kernels such as linear, polynomial, and radial basis function (RBF) are evaluated to capture potential non-linear decision boundaries.
- MLP: A flexible feedforward artificial neural network capable of modeling complex, non-linear relationships between inputs and the health state, representing a state-of-the-art approach for pattern recognition.

The preprocessed dataset is split into a training set (e.g., 70–80%) and a hold-out test set. Each model is trained on the training set via supervised learning. Hyperparameter tuning is performed using techniques like grid search or random search, optimized based on performance on a separate validation set or via cross-validation.

#### 2.3.3. Model Evaluation Metrics

The primary evaluation metric is the classification accuracy on the held-out test set. Accuracy is defined as the proportion of correctly classified samples:

(8) $$Accuracy=\frac{1}{N_{test}}\sum_{i=1}^{N_{test}}I\left({\hat{y}}_{i}=y_{i}\right)$$

where ${\hat{y}}_{i}$ is the predicted label, $y_{i}$ is the true label, and $I\left(\cdot \right)$ is the indicator function.

While accuracy provides a high-level summary of model performance, a more granular and informative analysis is achieved through the confusion matrix. This n × n matrix (where n is the number of classes, here n = 4 for health states A–D) is structured such that each row represents the true health state, and each column represents the predicted health state. The diagonal elements, known as True Positives (TP), indicate the number of instances correctly predicted for each corresponding state, with stronger models exhibiting higher values concentrated along this diagonal. The off-diagonal elements capture misclassifications: False Positives (FP) for a class appear in its corresponding column (excluding the diagonal), representing healthier states incorrectly alarmed as that class, while False Negatives (FN) for a class appear in its corresponding row (excluding the diagonal), indicating instances of that class missed by the model and predicted as other states.

In the context of AWE condition monitoring, analyzing these off-diagonal entries is crucial for identifying critical error types such as missed faults, where a true fault state is incorrectly predicted as healthier, posing a direct safety risk, and false alarms, where a normal or mildly degraded state is incorrectly predicted as a severe fault, potentially leading to unnecessary maintenance and operational disruption. Thus, the confusion matrix not only validates overall accuracy but also assesses the model’s diagnostic consistency and its balance between fault sensitivity and operational reliability, ensuring the selected intelligent monitoring agent is robust and trustworthy for real-world deployment [38].

## 3. Case Study: Application to a Laboratory-Scale AWE

This section details the specific application of the proposed hybrid framework to a concrete laboratory-scale AWE, providing the precise parameters and configurations that instantiate the general methodology.

### 3.1. System Description and Experimental Setup

**Figure 4:**
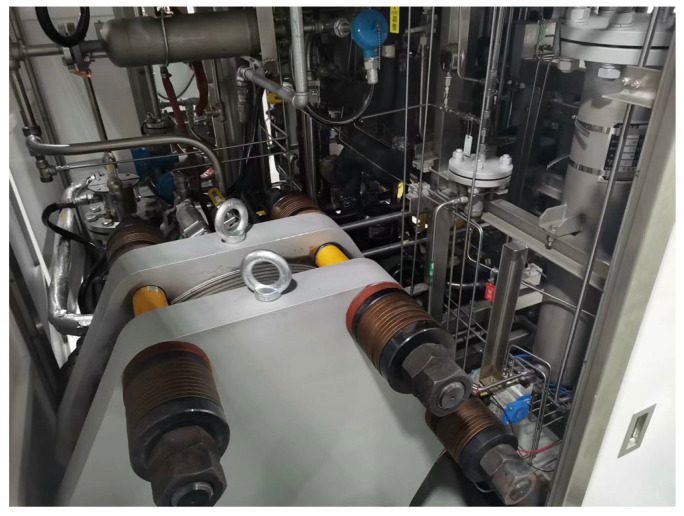
Photograph of the laboratory-scale AWE test rig used as the basis for this case study.

The subject of this case study is a rectangular, filter-press type AWE cell, a photograph of which is shown in Figure 4. This configuration is widely used in laboratory research for its well-defined geometry and ease of instrumentation.

All experiments were performed using a laboratory-scale, bipolar filter-press alkaline water electrolyzer possessing a nominal hydrogen production capacity of 2 Nm^3^/h. The test platform enables controlled adjustment of key operating parameters, including current density, electrolyte concentration, temperature, and pressure, providing a reliable experimental basis for model validation and condition-monitoring studies.

### 3.2. CFD Model Implementation

#### 3.2.1. Geometry and Mesh

**Figure 5:**
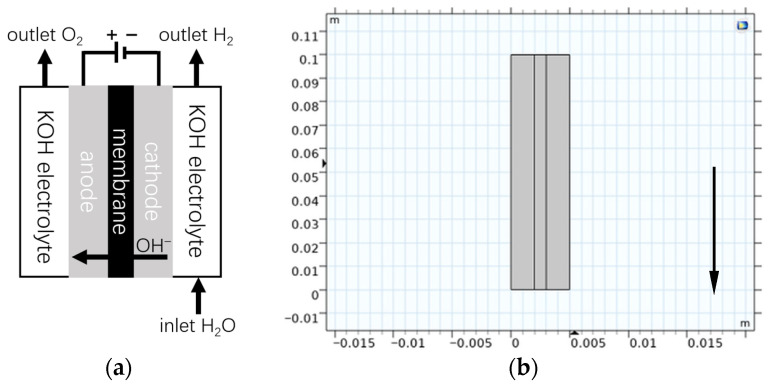
(**a**) Schematic diagram of an AWE electrolyzer; (**b**) 2D geometry model used in the CFD simulation (The gravity direction is indicated by the arrow).

The model geometry, depicted in Figure 5a, represents a simplified 2D cross-section of a symmetric AWE cell, consisting of anode and cathode compartments (2 mm width each) separated by a porous diaphragm (1 mm width), with an electrode height of 0.1 m (Figure 5b). The mesh consisted of approximately 25,000 rectangular elements. A mapped mesh was used in the bulk regions, and a boundary layer mesh with two layers (each 3 × 10^−5^ m thick) was applied at the electrode surfaces to capture the critical near-wall phenomena, following the strategy outlined in Section 2.2.2.

**Figure 6:**
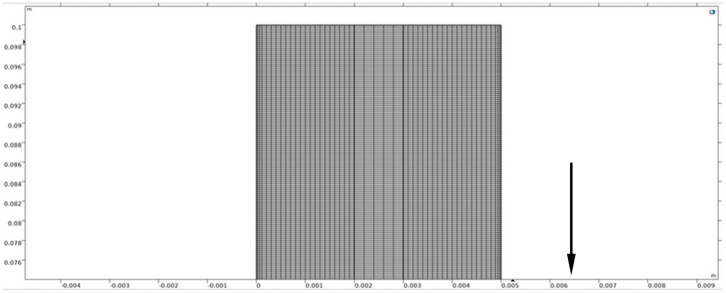
CFD mesh used in the simulation: mapped mesh in the bulk regions and two-layer boundary layer at electrode surfaces (layer thickness 3 × 10^−5^ m, total ~25,000 elements). (The gravity direction is indicated by the arrow).

The CFD mesh is shown in Figure 6, illustrating the mapped bulk mesh and boundary layer refinement at the electrode surfaces.

#### 3.2.2. Boundary Conditions, Assumptions, and Global Parameters

**Figure 7:**
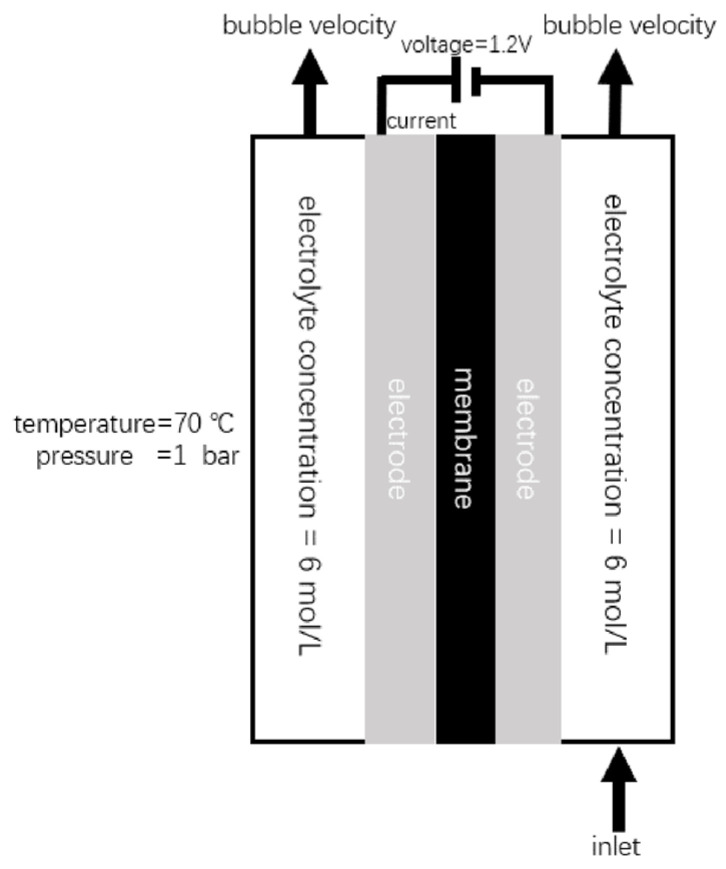
Schematic diagram of the initial and boundary conditions applied in the CFD model.

The model setup and key global parameters are summarized in Figure 7 and Table 3. The assumptions listed in Section 2.2 were applied. The electrolyte inlet velocity was set to 0.1 m/s for both compartments. The baseline operating conditions and material properties were defined as follows:

#### 3.2.3. Parametric Sweep for Dataset Creation

To generate the dataset for this specific cell, the parametric sweep was executed within the ranges detailed in Table 4, producing 625 data samples. The selected ranges are grounded in operational data from full-scale industrial AWE projects (e.g., China’s Da’an and Narisong green hydrogen plants), encompassing both normal and boundary conditions encountered in practice. This ensures that the resulting dataset provides a reliable and industrially relevant basis for constructing health indicators and benchmarking the MLP model. While wider ranges could further evaluate the robustness of different machine learning algorithms, the current scope is sufficient to demonstrate the framework’s validity under practical operating scenarios. Extensions to broader conditions are noted as a future research direction.

### 3.3. ML Model Configuration

The dataset for this study, comprising 625 labeled samples generated from the validated CFD model, was used for training and benchmarking the machine learning classifiers. All eight designated health indicators served as the input features. Prior to model training, these features were normalized using min–max scaling to eliminate the influence of differing physical units and scales. The output label was the discrete health state (A, B, C, or D), corresponding to the four predefined categories of system health.

The dataset exhibits a non-uniform distribution across health states, with 294 samples in state A, 199 in state B, 105 in state C, and 27 in state D. This imbalance reflects realistic AWE operating conditions, where severe fault states occur far less frequently than nominal or mildly degraded states. Given the low-dimensional and physics-constrained nature of the input feature space, the dataset size is sufficient for training compact machine learning models with controlled complexity.

Three distinct machine learning algorithms were implemented and configured to perform this multiclass classification task, with their key hyperparameters carefully selected to balance model capacity and generalization performance.

The polynomial regression (PR) model was employed as a transparent and interpretable nonlinear baseline to assess the capability and limitations of low-complexity parametric models in representing coupled degradation-related behavior in AWE systems. The model’s complexity was controlled by the polynomial degree, which was systematically varied from 2 to 6 during evaluation to identify an appropriate trade-off between model expressiveness and overfitting. By including PR in the benchmarking set, the study provides a reference for understanding the performance gains achieved by higher-capacity models when handling strongly coupled, physics-informed health indicators.

For the SVM classifier, a comprehensive kernel evaluation was conducted. The linear, polynomial, sigmoid, and radial basis function (RBF) kernels were tested to determine the most suitable mapping for the classification boundaries. The penalty parameter C, which controls the tolerance for misclassified samples, was tuned across a range of 1 to 7 to optimize the margin-error trade-off.

The MLP model was designed to capture the nonlinear relationships among the physics-informed health indicators while maintaining a compact architecture suitable for the available dataset size. The network consisted of 10 hidden layers with 8 neurons per layer, a configuration selected based on preliminary exploratory testing to balance model expressiveness and generalization performance. The Rectified Linear Unit (ReLU) activation function was employed in all hidden layers to mitigate vanishing gradient effects and accelerate convergence. Model training was performed for 500 epochs using the Adam optimizer. To reduce overfitting, L2 regularization was applied, with the regularization coefficient α tuned via grid search on a validation set.

All models were trained, validated, and tested using an identical data splitting protocol to ensure a fair comparison. Their performance was rigorously evaluated based on classification accuracy and a detailed analysis of the confusion matrix, as discussed in Section 4.

## 4. Results and Discussion

### 4.1. Validation of the Physics-Informed Dataset

The reliability of the proposed hybrid monitoring framework is fundamentally predicated on the fidelity of the underlying physics-based model and the physical consistency of the generated HIs. This section presents a two-tier validation strategy: first, the accuracy of the multiphysics CFD model is established through direct comparison with experimental measurements; second, the degradation-relevance and internal consistency of the HIs derived from the validated CFD model are systematically examined.

#### 4.1.1. CFD Model Validation Against Experimental Measurements

The predictive capability of the 2D multiphysics CFD model was first verified against experimental data obtained from a laboratory-scale AWE test rig. Experiments were conducted within the parameter ranges defined in Table 4. For each operating condition, the electrolyzer was operated until steady-state behavior was achieved, followed by continuous operation for a duration of 2 h to collect stable performance data. Recorded current signals were time-averaged, with transient disturbances filtered out.

**Figure 8:**
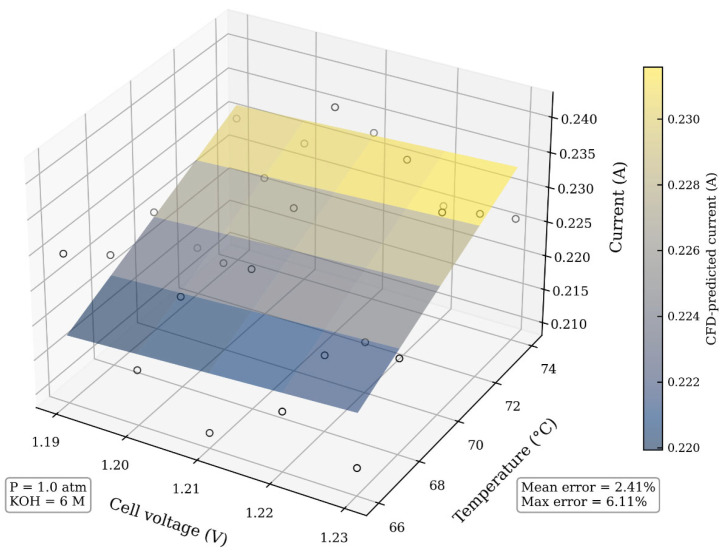
Comparison of CFD-predicted and experimental current.

As shown in Figure 8, the CFD-predicted current accurately reproduces the nonlinear dependence on cell voltage and operating temperature observed in the experiments. This current–voltage behavior represents an integrated response of electrochemical kinetics, ohmic losses, heat transfer, and gas–liquid mass transport, and is therefore a robust and practically accessible metric for model validation in operating AWEs. The quantitative comparison yields a mean relative error of 2.41% between the CFD results and experimental measurements, with a maximum deviation below 6.2%.

It should be noted that, due to the limited accessibility of internal measurements in operating electrolyzers, additional validation metrics such as local gas volume fraction, bubble size distribution, or spatial temperature fields could not be directly obtained without intrusive instrumentation. Accordingly, the present validation focuses on global steady-state electro-thermal performance, which is most relevant to the intended role of the CFD model as a physics-informed data generator for health indicator construction. Within this defined operational envelope, the close agreement observed confirms that the CFD model provides a physically consistent digital representation of the AWE’s steady-state behavior.

#### 4.1.2. Physical Consistency and Degradation-Relevance of the Generated HIs

Leveraging the validated CFD model, a comprehensive dataset of 625 samples was generated through the parametric sweep detailed in Section 3.2.3. This dataset provides a complete set of eight HIs for each simulated operating point, which are automatically labeled with a health state (A–D) based on the threshold rules in Table 2.

The physical plausibility of this dataset was critically examined by verifying the intrinsic, cause–effect relationships among the HIs, as dictated by fundamental electrochemistry and multiphase flow principles. For instance, analysis of data subsets with fixed voltage, temperature, and pressure confirmed that an increase in electrolyte pH (i.e., higher KOH concentration) leads to a corresponding increase in current density and Coulombic efficiency, while bubble velocity and relative power load decrease. This trend aligns perfectly with theoretical expectations: a higher concentration of OH^−^ ions enhances ionic conductivity and electrochemical reaction kinetics, thereby improving efficiency and reducing the gas evolution overpotential at a constant cell voltage. The consistent manifestation of such physico-chemical correlations across the entire dataset confirms that the CFD model operates as a faithful physics-informed data generator.

**Figure 9:**
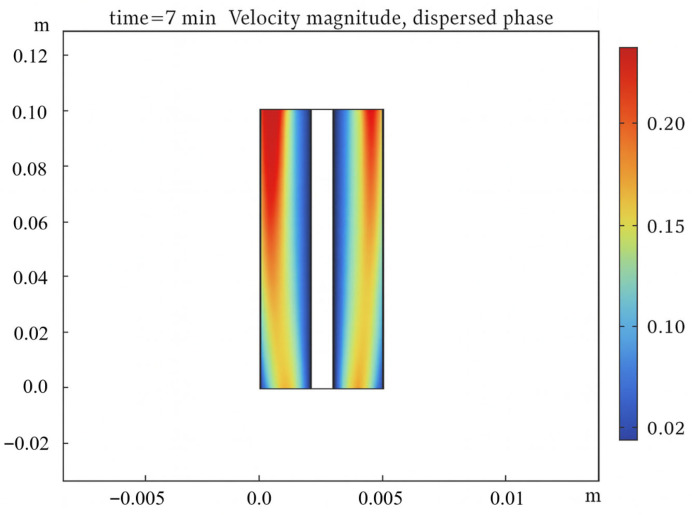
Contour of bubble velocity magnitude from CFD simulation.

Furthermore, the operational envelope covered by the dataset—including temperatures from 66 °C to 74 °C and pressures from 0.88 to 1.00 atm—effectively encompasses the precursor states of common faults such as mild overheating and under-pressure conditions. This demonstrates the model’s capability to simulate degradation-relevant operational scenarios, which is crucial for training a monitoring system aimed at early fault detection. While the steady-state CFD model cannot simulate catastrophic, transient fault events (e.g., explosive gas mixing), its strength lies in generating a physics-consistent knowledge base that captures the progressive shifts in operational parameters indicative of incipient degradation. Figure 9 visually exemplifies one such key HI, showing the spatial distribution of bubble velocity magnitude within the electrolyzer channel under a specific operating condition, a parameter directly linked to gas crossover risk and efficiency loss.

### 4.2. Performance Benchmarking of Intelligent Monitoring Algorithms

The core task of the intelligent monitoring agent is to perform rapid, accurate health state classification based on a subset of measurable inputs. Three candidate ML models-Polynomial Regression, SVM, and MLP-were rigorously benchmarked to identify the optimal surrogate for real-time inference.

#### 4.2.1. Polynomial Regression: An Interpretable Baseline

**Figure 10:**
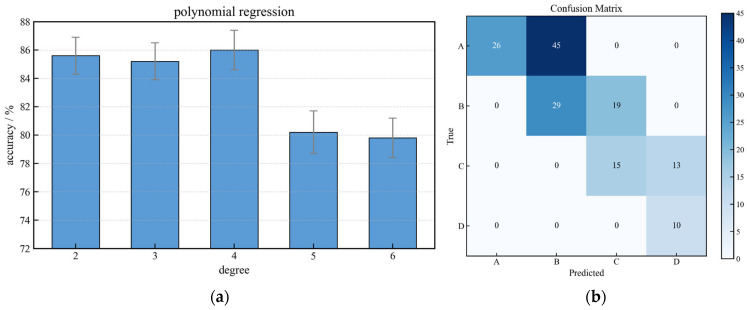
(**a**) Model accuracy vs. polynomial degree; (**b**) Confusion matrix for the optimal polynomial regression model (Degree = 4).

Polynomial regression served as an interpretable baseline model. Hyperparameter tuning focused on the polynomial degree (Figure 10a). The optimal accuracy of 85.94% was achieved with a 4th-degree polynomial. While this model captured the primary non-linear trends, its performance plateaued, reflecting a limited capacity to model the complex, high-dimensional decision boundaries between health states. The confusion matrix (Figure 10b) reveals a systematic bias towards underestimation (predicting a worse state than actual), leading to a high rate of false alarms. This conservative bias, though undesirable for operational efficiency, stems from the model’s simplicity and highlights the need for more sophisticated algorithms to balance sensitivity and specificity in industrial monitoring.

#### 4.2.2. SVM: Kernel Selection and Limitations

**Figure 11:**
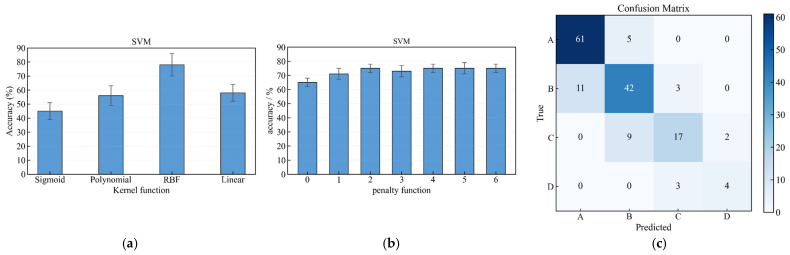
(**a**) Accuracy of SVM with different kernel functions; (**b**) Accuracy vs. penalty parameter C for the RBF kernel; (**c**) Confusion matrix for the optimized SVM model (RBF kernel, C = 2).

The SVM was evaluated with various kernel functions (Figure 11a). The RBF kernel yielded the best performance (78.13%), as it can create complex, non-linear boundaries. Further tuning of the penalty parameter C (Figure 11b) did not yield significant improvement, with optimal accuracy peaking at 76.22% for C = 2. The final confusion matrix (Figure 11c) shows that the SVM struggled with distinguishing between adjacent health classes (e.g., A vs. B, B vs. C), particularly in regions where the HI thresholds defined in Table 2 create subtle, multi-parametric boundaries. This suggests that while SVM is powerful for binary classification, its effectiveness for multi-class problems with interdependent, continuous-valued HIs is limited without extensive feature engineering or a much larger dataset.

#### 4.2.3. MLP: A High-Performance Intelligent Agent

**Figure 12:**
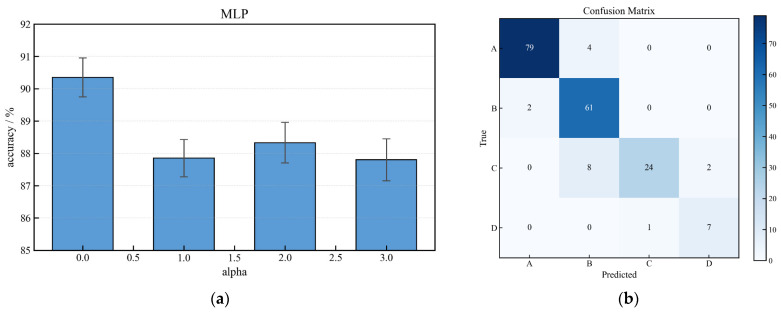
(**a**) MLP model accuracy vs. L2 regularization strength (*α*); (**b**) Confusion matrix for the optimized MLP model (*α* = 0.01).

Among the evaluated models, the MLP, a feedforward artificial neural network, demonstrated the best overall performance for this task. Tuning the L2 regularization hyperparameter *α* was crucial to prevent overfitting and ensure generalizability (Figure 12a). The best-performing MLP configuration (*α* = 0.01) achieved a peak classification accuracy of 90.43%.

The corresponding confusion matrix (Figure 12b) indicates strong performance across all health states. It is observed that the majority of misclassifications occur between neighboring health categories, which reflects the gradual and continuous nature of AWE performance degradation rather than sharply separated fault boundaries. Such behavior is consistent with the underlying physical degradation mechanisms.

In practical condition-monitoring applications, these localized misclassifications are acceptable when combined with temporal trend analysis and conservative decision thresholds, as they reduce the likelihood of overlooking severe fault conditions while supporting early-stage degradation detection. The MLP’s key strength lies in its ability to learn hierarchical, non-linear feature representations from the eight HIs, effectively approximating the complex mapping from operational parameters to the integrated health state defined by the proposed semi-quantitative evaluation framework.

#### 4.2.4. Class-Wise Performance Metrics and Safety Implications

In addition to overall classification accuracy, class-wise precision, recall, and F1-score were evaluated to provide a more comprehensive assessment of the monitoring algorithms, particularly for fault-related health states (C and D), which are critical in safety-sensitive AWE operation. Although the differences in overall accuracy among the models are moderate, the class-wise metrics reveal notable improvements in detecting fault conditions.

Precision reflects the reliability of fault alarms by quantifying the proportion of correctly identified fault states among all predicted fault instances, while recall measures the model’s ability to detect actual degradation or fault conditions. The F1-score provides a balanced metric that accounts for both false positives and false negatives.

As shown in Table 5, fault-related classes (C and D) exhibit lower recall than healthy states in general, highlighting the challenge of correctly identifying degraded conditions. Among the evaluated methods, the MLP demonstrates superior class-wise F1-scores for states C (0.814) and D (0.824), indicating a favorable balance between early fault detection and false-alarm avoidance. Notably, the higher recall for D class (0.875) compared to SVM (0.571) reduces the likelihood of false negatives, which pose the greatest safety risk in real-time AWE operation.

From an operational perspective, minimizing false negatives is essential, as misclassifying degraded or unsafe conditions as healthy could lead to hazardous scenarios. The MLP’s enhanced recall for fault-related states, combined with temporal trend analysis and conservative alarm thresholds, suggests improved robustness for real-time condition monitoring in safety-critical applications.

#### 4.2.5. Feature Importance Analysis Using SHAP

**Figure 13:**
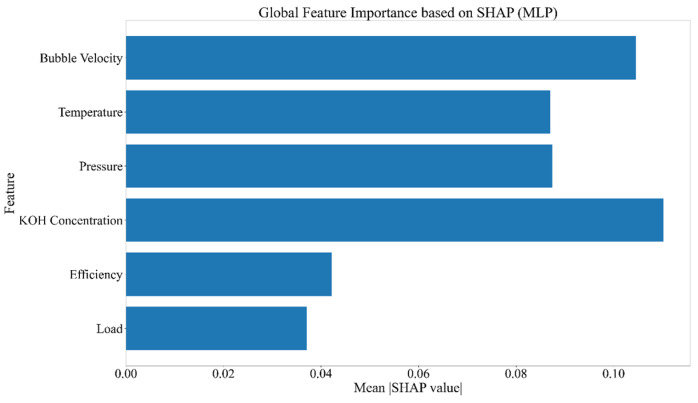
Global SHAP analysis of the MLP model showing the contribution of each health indicator to health state classification.

The global SHAP analysis of the MLP model (Figure 13) highlights KOH concentration and bubble velocity as the most influential health indicators, followed by pressure, temperature, efficiency, and load. The SHAP summary plot shows that variations in KOH concentration and bubble velocity strongly affect the predicted health state, indicating their high sensitivity to system changes. Pressure and temperature also contribute moderately, while efficiency and load have minor impact. These results provide insight into the model’s decision process and justify the selection of these indicators for effective health state classification.

### 4.3. Computational Efficiency and Practical Deployment Considerations

**Figure 14:**
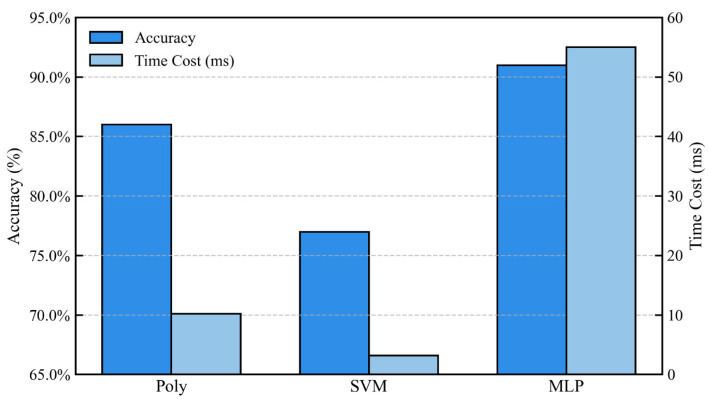
Comparative analysis of the three ML models in terms of classification ac-curacy and computational training time.

A critical consideration for real-time intelligent sensing is the trade-off between accuracy and computational latency. The training times for the final models were recorded: Polynomial Regression (9.91 ms), SVM (1.66 ms), and MLP (53.73 ms). While the MLP required the longest training time-a one-time offline cost-its inference time for a new data point is orders of magnitude faster (typically < 1 ms on modern hardware), making it perfectly suitable for real-time monitoring. Figure 14 synthesizes the accuracy-computational cost landscape. The MLP, occupying the high-accuracy region, is unequivocally the most suitable candidate for constructing the core intelligent monitoring agent. Its higher computational cost is justified by the paramount need for reliability in safety-critical applications like AWE operation. The model effectively translates the high-fidelity but slow physics-based understanding (CFD) into a fast, deployable software sensor for health state.

## 5. Conclusions

This study developed and validated a hybrid physics-informed ML framework for intelligent condition monitoring of AWEs, addressing challenges related to operational reliability. The work makes distinct contributions aligned with intelligent sensing and prognostics for complex industrial systems:

- Establishment of a Validated Physics-Based Foundation: A high-fidelity 2D multiphysics CFD model was developed and experimentally validated, serving as a credible “digital testbed.” This ensures that the generated dataset accurately captures key interactions within an AWE, providing a physically consistent knowledge base for training data-driven models.
- Physics-Informed Data Generation for AI Training: The CFD model generated a comprehensive labeled dataset reflecting the electrochemical–thermal–fluid interactions of an AWE. This approach addresses the scarcity of real-world fault data, providing a systematic basis for constructing health indicators and developing data-driven monitoring agents.
- Development of an Accurate Intelligent Monitoring Agent: Using the physics-informed dataset, an MLP model was identified as the optimal surrogate, achieving 90.43% accuracy in health state classification. This model operationalizes the hybrid framework, acting as a fast and interpretable software sensor that infers overall system health from accessible measurements.

The resulting methodology demonstrates how incorporating domain-specific physics enhances the interpretability and reliability of data-driven monitoring, offering a foundation for predictive maintenance that can improve safety, optimize maintenance schedules, and support more efficient green hydrogen production.

The current study is based on steady-state CFD simulations, and therefore the applicability of the framework to transient or highly dynamic operating scenarios, such as fluctuating renewable power inputs, has not yet been fully assessed. Future work will extend the approach to dynamic conditions using temporal feature analysis and sequence-based models (e.g., LSTM), conduct experimental validation of the HI system and MLP model on a physical AWE test rig, and explore integration with prognostic algorithms for RUL estimation. The incorporation of explainable AI techniques is also planned to enhance trust and facilitate adoption in practical industrial applications.

Overall, this work provides a structured and physically informed methodology for intelligent condition monitoring of AWEs, establishing a reliable framework for future extensions toward dynamic and real-world operating scenarios.

## Figures and Tables

**Table 1:** Common faults, phenomena, and consequences in AWEs.

Faults	Phenomena	Consequences
Gas Crossover	Increase in HTO (Hydrogen in Oxygen stream)/OTH (Oxygen in Hydrogen stream); Increased bubble velocity.	Risk of explosion if concentration exceeds threshold.
Electrode Corrosion	Decrease in electrode thickness; change in surface morphology.	Loss of efficiency, increased overpotential.
Hydrogen Leakage	Decrease in hydrogen content at outlet; detected by sensors.	Loss of production, potential explosion hazard.
Overheating	Abnormal increase in cell or stack temperature.	Accelerated degradation, thermal stress, potential shutdown.
Overpressure	Abnormal increase in system pressure.	Mechanical stress, potential for rupture or seal failure.
Control System Failure	Uncommanded changes or drift in key parameters (voltage, current, flow).	Unstable operation, potential to induce other faults.

**Table 2:** Criterion of AWE health condition (Voltage and current density are excluded from thresholding due to strong load dependence and redundancy under variable-power operation).

Health State	Threshold Ranges for Health State Classification					
	Temperature/°C	Pressure/atm	pH	Efficiency%	Bubble Velocity/m·s^−1^	Load/W
A	67 ≤ T < 71	0.96 ≤ P < 1.02	14.77 ≤ pH < 14.80	η ≥ 40	v ≤ 0.15	p ≥ 0.15
B	71 ≤ T < 73	0.93 ≤ P < 0.96	pH ≥ 14.80	35 ≤ η < 40	0.15 < v ≤ 0.18	0.10 ≤ p < 0.15
C	T < 67	0.90 ≤ P < 0.93	14.75 ≤ pH < 14.77	30 ≤ η < 35	0.18 < v ≤ 2.00	0.05 ≤ p < 0.10
D	T ≥ 73	P < 0.90	pH < 14.75	η < 30	v > 2.00	p < 0.05

**Table 3:** Global parameters and constants for the specific laboratory-scale AWE CFD model.

Parameter	Symbol	Value	Unit	Description
Compartment Width	W_H_2_, W_O_2_	2	mm	Width of H_2_ and O_2_ compartments
Diaphragm Width	W_sep	1	mm	Width of the separator
Cell Width	W_cell	5	mm	Total cell width (W_H_2_+W_sep+W_O_2_)
Electrode Height	H_elec	0.1	m	Height of the electrodes
Temperature	T	70	°C	Baseline operating temperature
Pressure	p_gas	1	atm	Baseline operating pressure
Bubble Diameter	d_bubble	50	µm	Assumed constant bubble diameter
Inlet Velocity	v_in	0.1	m/s	Electrolyte inlet velocity
Dispersion Factor (H_2_)	K_H_2_	5	m/s	H_2_ bubble dispersion coefficient
Dispersion Factor (O_2_)	K_O_2_	10	m/s	O_2_ bubble dispersion coefficient
Exchange Current (HER)	i0_ref_H_2_	100	A/m^2^	Reference exchange current density for HER
Exchange Current (OER)	i0_ref_O_2_	1	A/m^2^	Reference exchange current density for OER
Electrolyte Concentration	c_KOH	6	M	KOH molarity
Diaphragm Porosity	eps_sep	0.3	-	Separator porosity

**Table 4:** Parameter ranges for the CFD parametric sweep in the case study.

Parameter	Range	Step Size	Unit
Cell Voltage	1.19–1.23	0.01	V
Temperature	66–74	2	°C
Pressure	0.88–1.00	0.03	Atm
KOH Concentration	5.6–6.4	0.2	M

**Table 5:** Performance comparison of different machine learning models under different health states.

Model	Health State	Precision	Recall	F1-Score
Polynomial Regression	A	1.000	0.366	0.536
	B	0.392	0.604	0.475
	C	0.441	0.536	0.484
	D	0.435	1.000	0.606
SVM	A	0.847	0.942	0.884
	B	0.750	0.750	0.750
	C	0.739	0.607	0.667
	D	0.667	0.571	0.615
MLP	A	0.975	0.952	0.963
	B	0.836	0.968	0.897
	C	0.960	0.706	0.814
	D	0.778	0.875	0.824

## Data Availability

The authors confirm that the data underlying the results presented in this study, are available from the corresponding author upon reasonable request.

## Abbreviations

The following abbreviations are used in this manuscript:

AWE	Alkaline Water Electrolyzer
HIs	Health Indicators
CFD	Computational Fluid Dynamics
ML	Machine Learning
MLP	Multilayer Perceptron
SVM	Support Vector Machine
PHM	Prognostics and Health Management
CBM	Condition-Based Maintenance
DT	Digital Twin
RES	Renewable Energy Source(s)
HER	Hydrogen Evolution Reaction
OER	Oxygen Evolution Reaction
RBF	Radial Basis Function
SHAP	SHapley Additive exPlanations
